# Comparative Analysis of Genetic Determinants Encoding Cadmium, Arsenic, and Benzalkonium Chloride Resistance in *Listeria monocytogenes* of Human, Food, and Environmental Origin

**DOI:** 10.3389/fmicb.2020.599882

**Published:** 2021-01-14

**Authors:** Tereza Gelbicova, Martina Florianova, Lucie Hluchanova, Alžběta Kalova, Kristýna Korena, Nicol Strakova, Renáta Karpiskova

**Affiliations:** ^1^Department of Microbiology and Antibiotic Resistance, Veterinary Research Institute, Brno, Czechia; ^2^Faculty of Veterinary Hygiene and Ecology, University of Veterinary and Pharmaceutical Sciences Brno, Brno, Czechia

**Keywords:** *Listeria*, comparative genomics, cadmium, arsenic, benzalkonium chloride, resistance, mobile genetic elements

## Abstract

Environmental adaptation of *Listeria monocytogenes* is a complex process involving various mechanisms that can contribute to their survival in the environment, further spreading throughout the food chain and the development of listeriosis. The aim of this study was to analyze whole-genome sequencing data in a set of 270 strains of *L. monocytogenes* derived from human listeriosis cases and food and environmental sources in order to compare the prevalence and type of genetic determinants encoding cadmium, arsenic, and benzalkonium chloride resistance. Most of the detected genes of cadmium (27.8%), arsenic (15.6%), and benzalkonium chloride (7.0%) resistance were located on mobile genetic elements, even in phylogenetically distant lineages I and II, which indicates the possibility of their horizontal spread. Although no differences were found in the prevalence of these genes between human and food strains, they have been detected sporadically in strains from the environment. Regarding cadmium resistance genes, *cadA1C1_*Tn*5422* predominated, especially in clonal complexes (CCs) 121, 8, and 3 strains. At the same time, *qacH_*Tn*6188*-encoding benzalkonium chloride resistance was most frequently detected in the genome of CC121 strains. Genes encoding arsenic resistance were detected mainly in strains CC2 (located on the chromosomal island LGI2) and CC9 (carried on Tn*554*). The results indicated a relationship between the spread of genes encoding resistance to cadmium, arsenic, and benzalkonium chloride in certain serotypes and CCs and showed the need for a more extensive study of *L. monocytogenes* strains to better understand their ability to adapt to the food production environment.

## Introduction

*Listeria monocytogenes* is a facultative intracellular pathogen and at the same time ubiquitous saprophytic bacterium, which can also be isolated from the soil, water, and vegetation (Gray et al., 2006). *L. monocytogenes* is also known for its ability to persist in the food-processing environment. However, unique properties that would be entirely responsible for the persistence of *Listeria* have not yet been revealed (Carpentier and Cerf, 2011). *L. monocytogenes* can be considered a genetically heterogeneous species. Its isolates have been classified into a number of clonal complexes (CCs) with specific characteristics and frequency of occurrence. At present, the classification of *L. monocytogenes* into hypervirulent and hypovirulent clones is also used. The hypervirulent clones include strains of CC1, CC2, CC4, and CC6, which are mainly associated with human and ruminant listeriosis cases, and the hypovirulent clones, typically containing truncated internalin A, include strains of CC121 and CC9, which are mainly associated with the occurrence and persistence in food-processing environments (Maury et al., 2019).

The cadmium and arsenic resistance encoded by different genetic determinants often located on mobile genetic elements was one of the earliest environmental adaptations observed in *L. monocytogenes* (Parsons et al., 2019). The mechanisms of cadmium resistance known thus far include efflux pumps encoded by the following genes: *cadA1C1*, which are commonly located on the plasmid-carried transposon Tn*5422*; *cadA2C2* first described on plasmid pLM80; *cadA3C3* carried on the integrative and conjugative element (ICE); and *cadA4C4* located on Listeria Genomic Island 2 (LGI2) (Parsons et al., 2020). Even though cadmium resistance in *Listeria* can often be spread by horizontal gene transfer, arsenic resistance in *L. monocytogenes* is encoded mainly chromosomally by the *arsR1D2R2A2B1B2* cassette carried together with *cadA4C4* on LGI2 (Kuenne et al., 2013; Parsons et al., 2019). The insertion of mobile element LGI2 into the chromosome is likely mediated by phage integrase (Kuenne et al., 2013). The presence of this cassette of arsenic resistance genes on plasmids has been found occasionally in *L. monocytogenes* (Hingston et al., 2019). The *arsCBADR* operon located on Tn*554* transposon may also be involved in arsenic resistance in *L. monocytogenes* (Kuenne et al., 2013; Lee et al., 2017).

Arsenic resistance encoded by genes carried on LGI2 is primarily associated with its occurrence in *L. monocytogenes* strains of 4b serotype, especially in the hypervirulent clones CC1, CC2, and CC4 (Lee et al., 2017). Conversely, in strains of serotypes 1/2a, 1/2b, and 1/2c, the presence of LGI2 is rarely reported (Lee et al., 2017; Hingston et al., 2019). However, underlying mechanism of the effect of arsenic resistance genes or other genes of genomic island LGI2 on *L. monocytogenes* virulence remains to be elucidated. The only described mechanism demonstrating the effect of genes encoding heavy metal resistance on *L. monocytogenes* virulence is the *cadC3* gene, which, in addition to *cadA3* regulation, suppresses *lspB* gene expression and thus promotes *Listeria* survival in macrophages (Pombinho et al., 2017).

Conversely, cadmium resistance is more commonly described in *L. monocytogenes* strains of serotypes 1/2a and 1/2c isolated from food and food-processing environment (Mullapudi et al., 2008; Ratani et al., 2012), although *Listeria* is not exposed to any obvious selection pressure related to cadmium-related exposure in food-processing plants. In Northern Ireland, cadmium resistance was detected more frequently in repeatedly isolated *L. monocytogenes* from milk and non-dairy products than in sporadic strains obtained from eight different producers (Harvey and Gilmour, 2001). Similarly, the occurrence of different variants of resistance genes not only to cadmium, but also to arsenic, has been reported in persistent strains of *L. monocytogenes* CC14 and CC121 isolated from a rabbit meat-processing plant in Italy (Pasquali et al., 2018). The possible effect of these genes on the persistence of *L. monocytogenes* in food-processing plants has not yet been confirmed.

The resistance of *L. monocytogenes* to cadmium often correlates with the resistance to quaternary ammonium compounds, such as benzalkonium chloride, widely used for disinfection in the food industry (Mullapudi et al., 2008; Ratani et al., 2012). The benzalkonium chloride resistance is one of the factors that could contribute to the persistence of *Listeria* in food-processing facilities. The benzalkonium chloride resistance in *L. monocytogenes* is mediated by the activity of various efflux pumps encoded by the *bcrABC* cassette carried on plasmid pLM80 (Elhanafi et al., 2010), by *qacH* gene chromosomally located on the Tn*6188* transposon (Müller et al., 2013), *qacA* and *qacC* genes carried by plasmids (Xu et al., 2014; Hurley et al., 2019), *emrE* gene located chromosomally on LGI1 (Kovacevic et al., 2015), and *emrC* first described on plasmid pLMST6 (Kremer et al., 2017). In addition to efflux pumps carried on mobile genetic elements, the tolerance of *L. monocytogenes* to benzalkonium chloride can also be affected by the chromosomal multidrug efflux pump MdrL (Jiang et al., 2019). The plasmid-carried *bcrABC* cassette and the *cadA1* and *cadA2* genes are also found in non-pathogenic *Listeria* species (e.g., *Listeria innocua*, *Listeria welshimeri*) commonly detected in food-processing plants, which can become sources of these genes for *L. monocytogenes* (Katharios-Lanwermeyer et al., 2012; Korsak et al., 2019).

Even though the environment is burdened by anthropogenic activities (industry, agriculture), information on the occurrence of genetic determinants encoding resistance to heavy metals in *L. monocytogenes* strains isolated from environmental samples is missing up to now. Heavy metals are found in disinfectants, soil fertilizers, and livestock feeding and are recognized as environmental pollutants. Therefore, bacterial populations, with genetic determinants conferring resistance to heavy metals, can be found in the gut microbiota of intensively reared farm animal species. The pollution of soils with heavy metals is variable, virtually inextricable, and can contribute to the contamination of water sources (Doležalová Weissmannová and Pavlovský, 2017). At present, the differences between the occurrence of resistance genes to heavy metals such as arsenic and cadmium or quaternary ammonium compounds in *L. monocytogenes* clones isolated from various sources (humans, food, and environment) are not fully understood. In the Czechia, to date no study focused on the occurrence of genetic determinants encoding resistance to heavy metals and disinfectants in *L. monocytogenes* has yet been performed. Therefore, the aim of the present study was to carry out a comparative analysis of the occurrence and type of genetic determinants encoding cadmium, arsenic, and benzalkonium chloride resistance in *L. monocytogenes* from various sources, focusing on their clonal determination and origin of isolation.

## Materials and Methods

### Tested Strains of *L. monocytogenes*

A total of 270 *L. monocytogenes* strains isolated from food (*n* = 106), mainly ready-to-eat food (*n* = 92), including strains from meat (*n* = 45), dairy (*n* = 23), and fish (*n* = 6) products; delicatessen (*n* = 12); fresh vegetables (*n* = 6); raw meat (*n* = 6); and frozen vegetables (*n* = 8), from clinical cases of human listeriosis (*n* = 132) and the environment (*n* = 32) were collected between 2010 and 2020. Strains from the environment were obtained mainly from mud on the banks of surface water sources (*n* = 17), rotting vegetation (*n* = 5), soil (*n* = 5), surface water (*n* = 2), algae (*n* = 1), and moss (*n* = 2). Only one strain per each human listeriosis outbreak was included in the analysis, food strains originated from different producers. The strains were selected to reflect the proportions of *L. monocytogenes* CCs in humans and in food in the Czechia (Tomáštíková et al., 2019). All available environmental strains stored in the cryobank at the VRI in Brno, classified into serogroups by the matrix-assisted laser desorption ionization–time of flight mass spectrometry method, were analyzed (Koudelka et al., 2018). Strains of serotype 1/2a (*n* = 136) belonged to 24 CCs and 31 STs: CC7 (*n* = 7), CC8 (*n* = 31), CC11 (*n* = 1), CC14 (*n* = 5), CC18 (*n* = 4), CC19 (*n* = 2), CC20 (*n* = 5), CC21 (*n* = 5), CC26 (*n* = 3), CC29 (*n* = 3), CC31 (*n* = 1), CC37 (*n* = 9), CC101 (*n* = 4), CC121 (*n* = 14), CC124 (*n* = 2), CC155 (*n* = 8), CC193 (*n* = 1), CC200 (*n* = 1), CC204 (*n* = 6), CC398 (*n* = 1), CC403 (*n* = 3), CC415 (*n* = 1), CC451 (*n* = 16), and CC475 (*n* = 3). Strains of serotype 1/2b (*n* = 40) belonged to five CCs and nine STs: CC3 (*n* = 13), CC5 (*n* = 7), CC87 (*n* = 18), CC195 (*n* = 1), and CC288 (*n* = 1). Strains of serotype 4b (*n* = 79) belonged to five CCs and nine STs: CC1 (*n* = 27), CC2 (*n* = 21), CC4 (*n* = 3), CC6 (*n* = 26), and CC315 (*n* = 2). Strains of serotype 1/2c (*n* = 15) belonged to CC9. Detailed information about CCs and STs of strains isolated from various sources is mentioned in the Supplementary Table.

### Whole-Genome Sequencing

Genomic DNA was extracted using the Blood and Tissue kit according to the manufacturer’s instructions (Qiagen, Germany). Preparation of DNA libraries by Nextera XT DNA Library Preparation Kit (Illumina) and sequencing on the Illumina platform were carried out externally using MiSeq (*n* = 154) and NextSeq (*n* = 116) sequencers.

### Whole-Genome Sequencing Data Quality Assessment

Quality of the reads was checked for all fastq files using FastQC in Unipro UGENE v. 36 (Okonechnikov et al., 2012). Phred score greater than 30 was set as short-reads set quality criteria. Average depth coverage greater than 30× was required. The genome size (between 2.8 and 3.1 Mb), consistent with *L. monocytogenes* parameters, was considered as assembly quality criteria.

### Genome Analysis

The raw sequence data were assembled using the Velvet assembler version 1.1.04 integrated in Ridom SeqSphere^+^ software (version 6.0.2; Ridom GmbH, Münster, Germany) using optimized k-mer size and coverage cutoff values based on the average length of contigs with >1000 bp; reads were trimmed at their 5’ and 3’ ends until an average base quality of 30 was reached in a window of 20 bases. The nucleotide sequences of selected benzalkonium chloride, cadmium, and arsenic resistance genes were downloaded from the NCBI database (Supplementary Table). The presence of the monitored genes was detected using Ridom SeqSphere^+^ software. The analysis was performed at default setting, i.e., reference sequence identity was at least 90%, and base sequence identity with reference sequence was 99%. The presence of plasmids was searched for with the PlasmidFinder v2.1 tool (Carattoli et al., 2014) at https://cge.cbs.dtu.dk/services/. The sequences of benzalkonium chloride, cadmium, and arsenic resistance genes were annotated by Prokka 1.14.5 software. BLAST (The Basic Local Alignment Search Tool) available at https://blast.ncbi.nlm.nih.gov/Blast.cgi was used to verify the presence of plasmids pLM80 (GenBank AADR01000010) and pLMST6 (GenBank LT732640), the presence of transposons Tn*6188* (GenBank HG329628), Tn*5422* (GenBank L28104) and Tn*554* (GenBank FR33648), and also the presence of the complete LGI2 sequence (GenBank CM001159) in the tested strains in which benzalkonium chloride, cadmium, and arsenic resistance genes were detected using Ridom SeqSphere^+^ and Prokka software.

## Results

### Distribution of Cadmium, Arsenic, and Benzalkonium Chloride Resistance Genes in *L. monocytogenes* From Various Sources

In *L. monocytogenes* strains isolated from food, in sequences obtained by whole-genome sequencing, resistance genes to cadmium (36.8%), arsenic (23.6%), and benzalkonium chloride (9.4%) were detected more frequently in comparison with strains isolated from human cases of listeriosis (Table 1). In strains isolated from the environment, cadmium, arsenic, and benzalkonium chloride resistance genes were present only occasionally (Table 1). Out of the *L. monocytogenes* strains obtained from the environment, cadmium and arsenic resistance genes were detected in a strain isolated from decaying vegetation, whereas cadmium resistance genes and simultaneously benzalkonium chloride resistance genes were found in a strain isolated from pond water. In both cases, the strains originated from locations used for recreational activities outside industrial and agricultural areas. However, no significant difference in the prevalence of these genes was observed between strains from food and human cases. The co-occurrence of genes encoding cadmium and benzalkonium chloride resistance was found in 13 (4.8%) *L. monocytogenes* strains isolated from food (*n* = 6), human cases of listeriosis (*n* = 6), and the environment (*n* = 1). Arsenic resistance genes were detected mainly on the chromosomal island LGI2 together with cadmium resistance genes (in 10% of strains) in strains from human listeriosis (*n* = 15), food (*n* = 11), and environment (*n* = 11).

**TABLE 1:** Occurrence of genes encoding resistance to benzalkonium chloride, cadmium, and arsenic in *L. monocytogenes* of various origin.

Origin of strains	No. of tested strains	No. of strains (%) carrying genes encoding resistance to		
		Benzalkonium chloride	Cadmium	Arsenic
Human	132	8 (6.1%)	35 (26.5%)	16 (12.1%)
Food	106	10 (9.4%)	39 (36.8%)	25 (23.6%)
Environment	32	1 (3.1%)	2 (6.3%)	1 (3.1%)
Total	270	19 (7.0%)	75 (27.8%)	42 (15.6%)

### Occurrence and Types of Genetic Determinants Encoding Cadmium, Arsenic, and Benzalkonium Chloride Resistance

Genetic determinants of *L. monocytogenes* resistance to heavy metals, especially to cadmium (27.8%), were more frequently detected in the tested strains than resistance genes to benzalkonium chloride (7.0%). Arsenic resistance genes were detected in the genome of 15.6% strains. Regarding the genes encoding cadmium resistance, the *cadA1C1* genes carried on the Tn*5422* transposon predominated in the tested strains (17.8%), followed by the *cadA4C4* genes (10.0%) located on the LGI2 chromosomal island together with the genes encoding arsenic resistance (Table 2). In most strains positive for the *cadA1C1* (42/48) genes, Tn*5422* was typically carried on plasmids. In two strains, isolated from a clinical case of listeriosis and from a meat product, belonging to CC124, Tn*5422* was located on the chromosome. In four strains, the location of Tn*5422* could not be found according to the obtained sequencing data (Supplementary Table). The *cadA2C2* genes were detected in parallel with the *bcrABC* cassette on plasmids in 1.1% of the tested strains. The *cadA3C3* genes were not detected in any of the tested *L. monocytogenes* strains. Regarding genetic determinants encoding arsenic resistance, the *arsCBADR* operon located on the Tn*554* transposon (5.6%) was identified in the genome of the tested strains in addition to LGI2. The analysis of the obtained sequences revealed chromosomal location of the Tn*554* transposon in the tested strains.

**TABLE 2:** Distribution of genes encoding resistance to benzalkonium chloride, cadmium, and arsenic in tested *L. monocytogenes* strains.

Resistance to	Gene	Localization	No. (%) of positive strains	Origin of strains	Serotype	CCs (no. of positive strains)
Benzalkonium chloride	*qacH*	Transposon_Tn*6188*	11 (4.1%)	Human, food, environment	1/2a 1/2c 4b	CC121 (8); CC101 (1) CC9 (1) CC2 (1)
		Chromosome	4 (1.5%)	human, food	1/2a 1/2b	CC8 (1); CC20 (1); CC121 (1) CC3 (1)
	
	*qacA*	Plasmid	ND	ND	ND	ND
	
	*qacC*	Plasmid	ND	ND	ND	ND
	
	*emrE*	Chromosome_LGI1	ND	ND	ND	ND
	
	*emrC*	Plasmid_pLMST6	1 (0.4%)	Food	1/2a	CC8 (1)
	
Cadmium	*bcrABC cadA2C2*	Plasmid_pLM80	3 (1.1%)	Human, food	1/2a 1/2b	CC204 (2) CC288 (1)
	
	*cadA1C1*	Transposon*_*Tn*5422*	48 (17.8%)	Human, food, environment	1/2a	CC121 (13); CC8 (11); CC124 (2); CC21 (1); CC31 (1); CC155 (1); CC193 (1)
					1/2c	CC9 (5)
					1/2b	CC3 (11); CC5 (1); CC195 (1)
	
	*cadA3C3*	Chromosome	ND	ND	ND	ND
	
Arsenic	*cadA4C4 arsA1A2B1B2D1D2R1R2*	Chromosome_LGI2	27 (10.0%)	Human, food, environment	1/2a 4b	CC204 (6); CC14 (1) CC2 (20)
	
	*arsCBADR*	Transposon_Tn*554*	15 (5.6%)	Human, food	1/2c	CC9 (15)

*ND, not detected; CCs, clonal complexes; LGI1, *Listeria* genetic island 1; LGI2, *Listeria* genetic island 2.*

Benzalkonium chloride resistance genes were carried almost exclusively on mobile genetic elements and were detected in 7.0% of the *L. monocytogenes* strains tested. Benzalkonium chloride resistance was most often encoded by the *qacH* gene located on the Tn*6188* transposon (4.1%). Four strains of *L. monocytogenes* isolated from humans (*n* = 3) and vegetables (*n* = 1) with the *qacH* gene located on the chromosome, which did not carry Tn*6188* in the genome (Table 2), belonged to four different CCs (Supplementary Table). In the *qacH* gene of these strains, an insertion of guanine at position 365 (365insG, Met124X) was detected in contrast to the reference sequence (Müller et al., 2013). Subsequently, by comparing with the *qacH* sequences available in the NCBI database, they were identified as 100% identical with *qacH* MK944277 of *L. monocytogenes* (NCBI, Blast). The occurrence of *bcrABC* genes (1.1%) and the *emrC* gene (0.4%) located on the plasmids was occasional. By comparing the obtained sequences, it was found that plasmids carrying the *bcrABC* cassette together with *cadA2C2* belonged to the originally described plasmid type pLM80 (Elhanafi et al., 2010), and the *emrC* gene was located on the plasmid pLMST6 (Kremer et al., 2017). Neither the *qacA* and *qacC* genes located on plasmids nor the *emrE* gene located on chromosomal island LGI1 was detected in the tested strains (Table 2).

### Comparison of Cadmium, Arsenic, and Benzalkonium Chloride Resistance Genes in Different *L. monocytogenes* Clones

The prevalence of cadmium resistance genes was comparable in strains from lineage I (serotype 4b, 1/2b) and lineage II (serotypes 1/2a, 1/2c), namely, 27.7 and 28.5%. It was similar in arsenic resistance genes, where the prevalence found in strains of lineage I (16.8%) was slightly higher compared to strains of lineage II (14.6%). No arsenic resistance genes were detected in any tested strain of serotype 1/2b. In contrast, benzalkonium chloride resistance genes were more frequently detected in lineage II strains (10.6%) compared to lineage I strains (2.5%), regardless of the origin of the strains.

**FIGURE 1:**
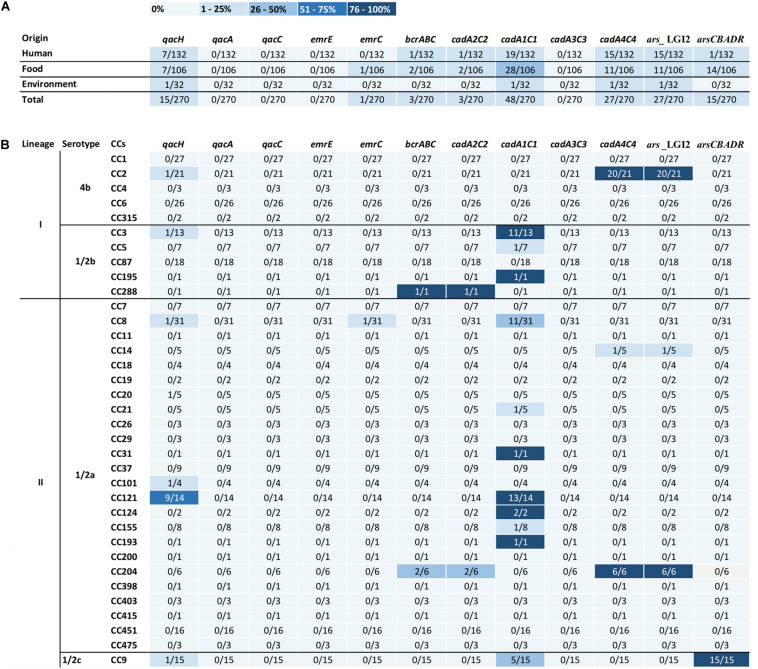
Heatmaps show the association of the occurrence of benzalkonium chloride, cadmium, and arsenic resistance genes **(A)** with *L. monocytogenes* origin and **(B)** with clonal complexes. CCs, clonal complexes. In columns for individual genes, the number of positive strains/number of tested strains of the specific CC, the intensity of blue color expresses the percentage.

Out of the detected 35 CCs of *L. monocytogenes*, resistance genes to benzalkonium chloride and heavy metals were detected only in strains belonging to 17 of them: CC8, CC14, CC20, CC21, CC31, CC101, CC121, CC124, CC155, CC193, CC204, CC288 (serotype 1/2a), CC9 (serotype 1/2c), CC2 (serotype 4b), CC3, CC5, and CC195 (serotype 1/2b). The prevalence of genes in strains belonging to different CCs is shown in Table 2. The prevalence of genes encoding benzalkonium chloride, cadmium, and arsenic resistance was typical of certain CCs. However, these genes were not found in the genome of any of the tested strains of the given CC (Figures 1A,B). These were, for example, cadmium resistance genes *cadA1C1*, which occurred mainly in the genome of strains CC121 (13/14; 93%), CC3 (11/13; 85%), CC8 (11/31; 35%), and CC9 (5/15; 33%). On the other hand, the genes of LGI2 chromosomal island encoding cadmium and arsenic resistance occurred mainly in CC2 strains (20/21; 95%), in all tested strains CC204 (6/6) and in one of the five tested strains CC14 (Figures 1A,B). The comparison of the detected chromosomal islands LGI2 in strain serotype 4b CC2 and serotype 1/2a CC204 and CC14 showed 100% identity. In addition to LGI2, two strains in CC204 isolated from ready-to-eat foods (ripened cheese and vegetables) simultaneously carried the *cadA2C2* and *bcrABC* genes located within plasmids. The resistance gene *arsCBADR* on Tn*554* was detected only in serotype 1/2c strains in the genome of all tested CC9 strains, isolated mainly from ready-to-eat meat products (*n* = 9), raw meat (*n* = 5), and also from human (*n* = 1).

## Discussion

### Distribution of Cadmium, Arsenic, and Benzalkonium Chloride Resistance Genes Among *L. monocytogenes* Strains From Humans, Food, and the Environment

Given the foodborne origin of listeriosis, it is not surprising that there was no significant difference in the prevalence of the monitored resistance genes to heavy metals and benzalkonium chloride between human and food strains collected in the Czechia. Twenty-three of 35 CCs (66%) tested in this study belonged to both strains of human and food origin. Environmental strains also belonged to the same CCs (9/13, 69%) detected (Supplementary Table). However, it was noteworthy that the tested strains isolated from the environment (e.g., soil, water), in which selection pressure could be expected due to anthropogenic activity, rarely carried genes of resistance to cadmium (6.3%) and arsenic (3.1%). One of the serotype 1/2a strains, CC204, isolated from decaying vegetation carried arsenic and cadmium resistance genes in LGI2. Another strain isolated from pond water belonged to CC121 and carried the resistance genes to cadmium *cadA1C1* located on Tn*5422* and at the same time the benzalkonium chloride resistance gene *qacH* on Tn*6188*. Both strains were isolated from sites without a direct industrial or agricultural burden. The rare detection of genetic determinants encoding heavy metal resistance in *L. monocytogenes* from the environment of the Czechia in this study may be due to the limited number of available strains tested in this group and the related low variability of CCs.

At present, there is a lack of studies to compare the occurrence of cadmium and arsenic resistance genes in *L. monocytogenes* in relationship to the source and country of origin. However, the prevalence of genes encoding resistance to cadmium (27.8%) and arsenic (15.6%) in *L. monocytogenes* strains isolated from humans, food, and the environment in the Czechia correlates with the high prevalence of heavy metal resistance described in studies from other countries (McLauchlin et al., 1997; Mullapudi et al., 2008; Xu et al., 2014; Lee et al., 2017). The higher proportion of cadmium resistance genes in comparison with arsenic resistance genes could be explained by a more frequent environmental exposure of *L. monocytogenes* to cadmium. *L. monocytogenes* can be commonly isolated from the environment of farm animals. Zhen et al. (2020) reported in their study that continuous application of manure over 15 years has significantly increased the concentration of cadmium in the soil (by 230%), while the concentration of arsenic was relatively stable.

Regarding cadmium resistance genes, mainly *cadA1C1* genes, predominated (17.8% of strains), and conversely, chromosomally located *cadA3C3* genes were not detected in any strain, which is in accordance with other studies (Ratani et al., 2012; Lee et al., 2013; Xu et al., 2014; Zhang et al., 2015). Some studies reported that all benzalkonium chloride-resistant strains were also cadmium-resistant (Mullapudi et al., 2008; Ratani et al., 2012; Zhang et al., 2015). However, this coresistance cannot always be explained by the presence of the *bcrABC* cassette and *cadA2C2*, which are carried on the same plasmid. In our study, the co-occurrence of genetic determinants encoding resistance to cadmium and benzalkonium chloride was demonstrated in 4.8% strains and the plasmid carrying *bcrABC* cassette and *cadA2C2* operon in only 1.1% of strains isolated from different sources. In some strains, the phenotypic manifestation of resistance or tolerance to benzalkonium chloride could also be affected by the multidrug chromosomal efflux pump MdrL (Jiang et al., 2019), which was detected in the genome of all tested strains.

Benzalkonium chloride resistance genes in *L. monocytogenes* were detected in only 7% of the strains tested in this study compared to cadmium and arsenic resistance genes. The analysis of almost 3000 *L. monocytogenes* genomes in France has shown that some of the benzalkonium chloride resistance genes have been identified in 32.1% of strains obtained by surveillance of listeriosis (Maury et al., 2019). A high prevalence of benzalkonium chloride resistance in *L. monocytogenes* was also found in another study characterizing 100 *L. monocytogenes* strains from three meat and vegetable food-processing plants, with the occurrence of resistance genes in 62% strains, increasing to 73% among suspect persistent strains (Hurley et al., 2019). In the collection of *L. monocytogenes* from Switzerland and Finland, resistance to benzalkonium chloride was demonstrated in 12.3 and 10.6% of strains isolated mainly from food, but also from humans and wild bird feces (Meier et al., 2017). The lower prevalence of benzalkonium chloride resistance genes in *L. monocytogenes* strains isolated in the Czechia might have been caused by selection of the tested strains in different studies, but also by habits in using quaternary ammonium compounds as disinfection agents in food-processing plants in particular countries.

Regarding benzalkonium chloride resistance, Maury et al. (2019) in France most frequently detected the *qacH* gene (18.8%), followed by the *bcrABC* (8.2%), *emrC* (5%), *qacC* (0.1%), *emrE* (0.03%), and *qacA* (0.03%) genes. As part of the LiSEQ project (Listeria SEQuencing), in a collection of strains from the European Union derived from ready-to-eat foods, food animals, food-processing environments, and humans, the *qacH* gene was detected in almost the same proportions (in 18.5% of strains), similarly to *bcrABC* (5% strains) and the *emrE* and *qacA* genes (<1% of strains) (Painset et al., 2019). In *L. monocytogenes* strains originating in Switzerland and Finland, resistance to benzalkonium chloride was also most frequently encoded by the *qacH* gene, namely, in approximately 57% of strains (Meier et al., 2017). Our findings are consistent with the above study as in our study the *qacH* gene predominated in the tested group, despite the lower prevalence of benzalkonium chloride resistance genes in strains isolated from humans, food, and the environment in the Czechia (5.6%).

### Occurrence of Genetic Determinants Encoding Cadmium, Arsenic, and Benzalkonium Chloride Resistance Depending on Species Heterogeneity of *L. monocytogenes* Strains

The genes encoding cadmium, arsenic, and benzalkonium chloride resistance in the tested strains of *L. monocytogenes* were carried mainly on mobile genetic elements. The detection of specific resistance genes in phylogenetically distant lineages I and II is indicative of their potential horizontal spread (Table 2). However, the ability to resist or tolerate heavy metals and disinfectants could provide an advantage to certain *L. monocytogenes* serotypes and CCs in their spread. The results showed that rather than the source of *L. monocytogenes*, the occurrence of resistance genes to cadmium, arsenic, and benzalkonium chloride is associated with certain serotypes and CCs, the distribution of which may differ in various countries. A number of studies have reported a frequent occurrence of cadmium resistance genes and a sporadic occurrence of arsenic resistance genes in serotype 1/2a strains in comparison with serotype 4b strains (McLauchlin et al., 1997; Mullapudi et al., 2008; Lee et al., 2017). In the present study, the occurrence of cadmium resistance genes was similar in the strains of all serotypes tested: 1/2a (27.9%, 38/136), 1/2c (33.3%, 5/15), 4b (25.3%, 20/79), and 1/2b (32.5%, 13/40). In contrast, arsenic resistance genes occurred only rarely in serotype 1/2a strains (5.1%, 7/136) and were not detected in any strain of serotype 1/2b, which is in accordance with other studies (McLauchlin et al., 1997; Mullapudi et al., 2008; Ratani et al., 2012), despite the fact that they belong to the same evolutionary lineage as strains of serotype 4b. In this study, arsenic resistance genes were detected more frequently in serotype 1/2c strains (100%, 15/15) than in serotype 4b strains (25.3%, 20/79).

In the Czechia, *L. monocytogenes* serotype 1/2a, with frequent occurrence of CC8, has long prevailed not only in humans, but also in foods (Tomáštíková et al., 2019). In CC8 strains tested in the current study obtained from both clinical cases of listeriosis (*n* = 18) and food (*n* = 13), we demonstrated a relatively frequent occurrence (35.5%) of cadmium resistance genes *cadA1C1* located on the transposon Tn*5422* related to plasmid. On the other hand, in many European countries, strains of serotype 4b of CCs CC1, CC2, CC4, and CC6 predominated in clinical cases of listeriosis (Painset et al., 2019). In the genome of strains CC2 (84%), CC1 (35%), and CC4 (24%), but not in strain CC6, Lee et al. (2017) demonstrated the presence of LGI2 in which cadmium and arsenic resistance genes are located simultaneously. They reported that LGI2 shows significant content plasticity and is capable of transferring different accessory genes to diverse chromosomal locations. In our study, we demonstrated LGI2 mainly in the CC2 (20/21), associated with hypervirulence (Maury et al., 2019), but not in CC1 and CC4 strains. However, the effect of LGI2 on *L. monocytogenes* virulence has not yet been elucidated. For example, in the Czechia, within serotype 4b, CC6 strains predominate among clinical cases of listeriosis (Tomáštíková et al., 2019), in which this genetic island does not occur.

While in strains of serotypes 4b and 1/2a, the arsenic resistance genes were located on LGI2, all tested 1/2c strains of CC9 carried in the genome arsenic resistance genes on Tn*554* located on the chromosome. Our results are consistent with previous studies describing more frequent occurrence of arsenic resistance genes on Tn*554* in *L. monocytogenes* serotypes 1/2c and 1/2a (Kuenne et al., 2013; Lee et al., 2017). With the exception of one human strain 1/2c, the other strains of serotype 1/2c tested in this study (*n* = 14) were derived from food sources. Within serotype 1/2c, McLauchlin et al. (1997) reported more frequent cadmium resistance and less frequent arsenic resistance in strains derived from clinical cases of listeriosis in comparison with food strains. This could explain the high occurrence of arsenic resistance genes found in strains 1/2c tested in this study. In the Czechia, *L. monocytogenes* serotype 1/2c is very rarely associated with human listeriosis cases (Tomáštíková et al., 2019), and therefore, it was not possible to compare these findings.

It was noteworthy that all CC204 strains tested in this study carried LGI2 regardless of their origin. The tested group of strains CC204 (*n* = 6) included strains isolated from the environment, vegetables, and persistent strains from two cheese-processing plants (Supplementary Table). The occurrence of LGI2 has already been detected in CC204 strains (Hingston et al., 2019; Palma et al., 2020), as well as in strains CC14 and CC121 (Pasquali et al., 2018). However, in this study, LGI2 was detected in only one of the five CC14 strains isolated from fresh cheese and in none of the CC121 strains. In France, LGI2 has also been detected in persistent strains of *L. monocytogenes* CC204 derived from seafood-processing plants (Palma et al., 2020). Even though the role of LGI2 in *L. monocytogenes* persistence is not clear, current studies suggested that mobile genetic elements could improve *Listeria* survival rates in the environment of food-processing plants (Pirone-Davies et al., 2018; Palma et al., 2020).

In addition to cadmium resistance, more frequent resistance to benzalkonium chloride has been reported in lineage II strains (serotypes 1/2a and 1/2c) in a number of European studies (Meier et al., 2017; Pirone-Davies et al., 2018; Maury et al., 2019; Painset et al., 2019) and in the United States (Mullapudi et al., 2008; Ratani et al., 2012). In our study, benzalkonium chloride resistance genes were also detected more frequently in lineage II strains. On the other hand, in a Chinese study, benzalkonium chloride resistance was detected mainly in *L. monocytogenes* serotype 4b isolated from food, probably due to a very low number of resistant strains tested (Xu et al., 2014). The results of a number of studies suggested that the occurrence of benzalkonium chloride resistance genes is mainly associated with CC9 and CC121 (Meier et al., 2017; Maury et al., 2019). This is confirmed by the results of our study, where more than half of the tested CC121 strains (60%, 9/15) carried the *qacH* gene. In *L. monocytogenes* CC9, in which arsenic resistance predominated, benzalkonium chloride resistance genes were detected in 7% (1/15) of the strains tested. For strains of the CC121 CC, the results of this and other studies (Maury et al., 2019) also suggested coresistance to cadmium. With the exception of one strain isolated from a meat product, all *L. monocytogenes* CC121 tested in this study isolated from food, humans, and the environment carried *cadA1C1_*Tn*5422* (Supplementary Table).

Because of the fact that the majority of cadmium, arsenic, and benzalkonium chloride resistance genes are carried on mobile genetic elements with the possibility of horizontal spread, the occurrence of these genes is not always related to all strains of a particular *L. monocytogenes* CC, as shown by the results of the present study (Figures 1A,B). An example is the occurrence of the Emrc efflux pump carried on plasmid pLMST6, originally described for strains of serotype 4b CC6 (Kremer et al., 2017), and also for strains of serotype 1/2a CC8, CC31, and CC403 isolated from humans and food (Kropac et al., 2019). In contrast, in our study, a plasmid carrying the *emrC* gene was detected in a single strain of CC8 (0.4%) and in none of the tested strains CC31 and CC403 isolated from human listeriosis cases, food, and the environment.

## Conclusion

In the present study, no differences were found in the occurrence and type of genetic determinants encoding resistance to heavy metals such as cadmium and arsenic and the disinfecting agent benzalkonium chloride between strains isolated from human listeriosis cases and food. The higher prevalence of resistance genes, especially to cadmium, but also to arsenic, compared to benzalkonium chloride resistance genes may indicate a higher selection pressure of the environment due to heavy metal contamination and limited use of benzalkonium chloride for disinfection in the Czechia. The distribution of cadmium, arsenic, and benzalkonium chloride resistance genes carried mainly on mobile genetic elements showed affinity for specific CCs, regardless of the source of *L. monocytogenes*. This may be the reason for sporadic finding of heavy metal resistance genes in *L. monocytogenes* strains originating from the environment of the Czechia tested in this study. The obtained results point to the need for further research into the characteristics of *L. monocytogenes* isolated from environmental sources to understand the dissemination of specific clones throughout the food chain.

## Data Availability Statement

The datasets presented in this study can be found in online repositories. The names of the repository/repositories and accession number(s) can be found below: the European Nucleotide Archive (ENA) at EMBL-EBI under accession number PRJEB37840 (https://www.ebi.ac.uk/ena/data/view/PRJEB37840) and PRJEB40064 (https://www.ebi.ac.uk/ena/data/view/PRJEB40064).

## Author Contributions

All the authors read and agreed to the published version of the manuscript. TG and RK performed the concept and methodology of this study. TG wrote the manuscript. KK and NS extracted genomic DNA for WGS. MF, LH, and AK performed the bio-informatic analysis. All authors contributed to the article and approved the submitted version.

## Conflict of Interest

The authors declare that the research was conducted in the absence of any commercial or financial relationships that could be construed as a potential conflict of interest.
